# Progress in Pediatrics

**Published:** 1900-11-10

**Authors:** 


### Progress in Pediatrics.

**Prolapse of the Rectum.**—This condition is manifested by a tumour projecting from the anus, the form of the tumour varying in different cases. It is usually sausage-shaped, but may be conical, or globular, or pear-shaped, or sickle-shaped. The tumour is coated with mucous membrane, and may be from 6 to 75 centimetres in length. The inclusion of the peritoneum in the prolapse is an important complication from the operative point of view. C. G. Cumston[7] points out that the peritoneum passes down from the third sacral vertebra along the lateral surfaces of the rectum, detaches itself from the gut in a curved line, forming a horseshoe with its concavity directed backwards and upwards, and then passes forwards on to the bladder in the male and the uterus in the female. Therefore the terminal portion of the rectum—at least, for its lower two-thirds—is extraperitoneal. As the anus is about seven centimetres from the lowest part of the peritoneum, the prolapse must be of considerable size before the serous membrane can become insinuated between the two cylinders of gut forming the invagination. There is, unfortunately, no clinical indication as to whether the peritoneum is present in the tumour or not. If the part of the rectum with peritoneum attached is involved in the prolapsus, then the peritoneum must go with it, and will be found at the anterior part of the tumour. As a matter of experience, such an occurrence is rarely met with, a fortunate state of affairs, because otherwise the peritoneum would necessarily be opened whenever incision was practised as the treatment. Another possible complication to be borne in mind is the presence of small intestine in the invagination; and cases have been known where a knuckle of gut was strangulated in the anterior *cul-de-sac* of the invagination. The signs of the presence of small intestine in the invagination are (1) a gurgling sound when the tumour is reduced by manipulation; (2) on coughing, the anterior aspect of the prolapsed portion will swell (Gosselin); and (3) the orifice of the tumour is directed backwards towards the sacrum (Allingham). To the causes of *prolapsus recti* as ordinarily given—namely, fits of coughing, difficulty in passing the stools, and a defect in the curve of the sacrum, the writer adds another—namely, *infection*—produced by retained fæces or infective diarrhœa. The pathological changes induced in the wall of the intestine deprive it of its tonicity and render it lax. In older children the surgeon must further examine for the presence of a polypus, or an ulcer, or hæmorrhoids, or some other lesion of the rectum. The results of prolonged or repeated prolapse are that the mucous surface is covered with a bloody or purulent mucus, and may lose its epithelium and become ulcerated. Abscesses may form, polypus-like granulating growths may develop, and more or less severe hæmorrhage may take place. Secondary results may be incontinence of fæces, and marked irritability of the bladder. In the majority of cases, prolapse of the rectum will yield to medical treatment in the early stages. The judicious use of a rubber rectal plug to keep the prolapse reduced, cleanliness, and tonic treatment with the use of strychnine will give the best results. If there is a definite local lesion, apart from the prolapse, this must first be treated by the surgeon. When a prolapse has reached a certain stage, when it has become irreducible, or when it has become constricted by the sphincter ani and gangrene is imminent, resection of the prolapsed part is the method of choice. The various forms of operative treatment are described and discussed. Very successful results have been obtained in this affection, according to Hajeah,[8] by the use of ice tampous. Cone-shaped suppositories of ice, made in moulds, with a length of from 3 to 3½ inches, and a diameter at the base of from 1 to 1¼ inches, are used for this purpose. Each is enveloped in a piece of iodoform gauze, and pushed gradually and steadily up the centre of the prolapse until the whole is reduced, the ice and gauze being carried up with the bowel. A fresh suppository is introduced after every act of defalcation, and the tendency to prolapse will thus be steadily diminished. The beneficial result is due partly to the relief of venous congestion and of laxity of the mucous tissue, and partly to the increased contractility and tonicity of the muscular coat produced by the coldness of the ice.

**The Operative Treatment of Tuberculous Peritonitis.**—Augustus Caillé[9] states that the diagnosis of tuberculous peritonitis is based on the abdominal symptoms, such as distension, pain, disturbed bowel action, and the presence of fluid, and on the progressive loss of weight. In doubtful cases he recommends an exploratory laparotomy. The clinical varieties are (1) the chronic tuberculous, with ascites, (2) fibro-caseous tuberculous peritonitis, (3) fibro-adhesive tuberculous peritonitis, and to these he adds another, namely, (4) tuberculous peritoneal tumours, of which he has seen two examples. As regards the treatment he finds that medical measures are useless, and recommends early laparotomy. Even with this treatment he notes that mild abdominal symptoms often persist for a long time, along with evidences of catarrhal conditions elsewhere. If at the time of the operation there is also tuberculosis of the lungs or pleura, the ultimate results are unsatisfactory, although some improvement usually takes place. In the discussion which followed Caillé's paper, Dr. Rotch said that he had had a wide experience of primary tuberculous peritonitis, and the treatment was invariably laparotomy. He believed that a very large number of the cases were not only benefited but also cured.

**Lobar Pneumonia and Enteric Fever.**—In a clinical lecture on lobar pneumonia Marfau[10] discusses the differential diagnosis between it and typhoid fever. In the early stages the physical signs of both may be absent and the temperature curve is not diagnostic, while diarrhœa is frequently present in both affections. In pneumonia, however, neither cold baths nor quinine will lower the temperature as in typhoid fever. When in doubt, one must examine the blood, but not for the Widal reaction. This test will not be of use in children unless one can be absolutely sure, when it is present, that the child has not recently had typhoid fever, because the reaction persists for many months with great intensity. Its presence, then, does not exclude pneumonia. Further, this reaction, in the case of children, appears at a later stage of typhoid fever—namely, after the fifteenth day, by which time there can be little doubt as to whether pneumonia had been present. There is, however, a blood test which is valuable, and is more easily carried out than that by agglutination. This is the appearance, in the case of pneumonia, of a very distinct fibrinous reticulum of the blood, which can be seen with the microscope at the time of coagulation. In normal blood and in the blood of typhoid fever these filaments are not visible. The reticulum may take some time to form—even half-an-hour—but its presence is diagnostic of pneumonia.

[7] Annals of Surg., March, 1900. [8] Rev. Mens. des Malad. de l'Enfance and Ep. B.M.J., March 10, 1900. [9] Arch. of Ped., June, 1900. [10] La Sem. Medic., Jan. 24, 1900.

